# A key to the Mexican and Central America Genera of Anthonomini (Curculionidae, Curculioninae)

**DOI:** 10.3897/zookeys.260.3989

**Published:** 2013-01-18

**Authors:** Macotulio Soto Hernández, Robert W. Jones, Pedro Reyes Castillo

**Affiliations:** 1 Instituto de Ecología, A.C. Carretera antigua a Coatepec 351, El Haya, Xalapa 91070, Veracruz. México; 2 Facultad de Ciencias Naturales, Universidad Autónoma de Querétaro, Juriquilla 76230, Querétaro, México

**Keywords:** Coleoptera, Curculionidae, Weevils, dichotomous key

## Abstract

Presently the only keys available for identification of genera of Anthonomini are limited to those of the United States of America and Canada. A dichotomous key is presented to identify all genera of Mexican and Central American Anthonomini. Previous keys do not include the genera *Achia*, *Botanebius*, *Loncophorus*, *Loncophorellus* and *Melexerus*. A brief synopsis is given for each genus and photographs of representative species are included.

## Introduction

The family Curculionidae of Mexico and Central America is rich in species (Anderson and O’Brien 1996). Although general keys to the subfamilies of Curculionidae are available (Morrone 2000, Anderson 2002, Marvaldi and Lanteri 2005), the absence of regional keys to genera limit the study of Curculionidae in Mexico and Central America (Anderson and O’Brien 1996). Identification of some genera of weevils is possible through the use of the Biologia Centrali-Americana (Sharp and Champion 1911, Champion 1903, 1910) and by using keys for the United States of America and Canada (Kissinger 1964, Anderson 2002); however, material in the Biologia Centrali-Americana is notably outdated, keys are not always presented, and the North American keys do not include a significant portion of the Mexico and Central American genera, moreover, the taxonomic status of some genera has changed and new genera have been described and recorded for the region.

**Figures 1–3.**
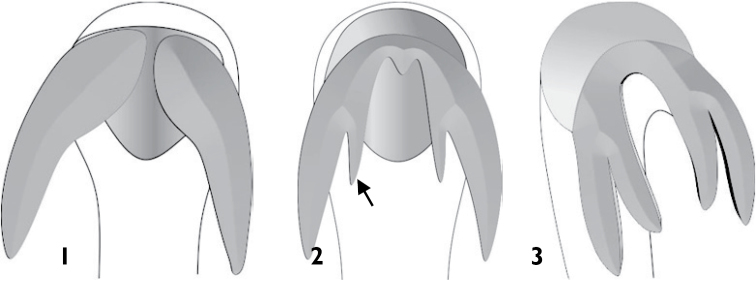
Tarsal claws of Anthonomini species: **1** simple tarsal claws **2** tarsal claws with an acute tooth **3** tarsal claws with a stout tooth.

Anthonomini is a tribe within the subfamily Curculioninae of the Curculionidae (*sensu* Alonso-Zarazaga and Lyal 1999). The tribe is one of the most diverse and complex of the family containing more than 800 described species within 43 genera, 24 of which are from the New World and 17 of these from México and Central America (Alonso-Zarazaga and Lyal 1999). The following characters will aid in placing species in this tribe collected in Mexico and Central America: rostrum free, not received into ventral channel, more or less cylindrical in cross section (Figs 18–24), longer than pronotum; antenna with scape not or just reaching the anterior margin of the eye; eyes nearly round; pronotum wider than long, narrowed in front, lacking postocular lobes (Figs 31–32); mesepimeron not ascended and not visible in dorsal view; elytral variable, generally wider at the base than the pronotum (Figs 27 and 29), striae punctured; pygidium covered by elytra; anterior coxae more or less equidistant from anterior and posterior margins of prosternum; sutures of abdominal ventrites straight and deep, except the first, which is less deeply impressed; tibia with a tooth at apex, usually larger on pro- and mesotibia; tarsi with claws free at base and with basal process or tooth (simple in *Epimechus* and *Brachyogmus* (Fig. 1)) (Dietz 1891, Kissinger 1964, Burke 1976 and Anderson 2002). Several species of Anthonomini are superficially similar to *Smicronyx* (Smicronychini), *Phyllotrox* (Derelomini) or *Tychius* (Tychiini). These taxa can be distinguished from Anthonomini by the following combination of characters: *Smicronyx* have claws connate at base and pronotum with postocular lobes. *Phyllotrox*, femur with ventral margin simple, lacking tooth; procoxae closer to posterior margin than to anterior margin of prosternum. *Tychius*, suture between ventrites 2 and 3 markedly extended posterolaterally, reaching or passing suture between ventrites 3 and 4 (Tanner 1966 and Kissinger 1964).

The host plants or plant associates of Anthonomini represent more than 35 families, including many species of agricultural importance. Two of the best-known pest species are the cotton boll weevil *Anthonomus grandis* Boheman and the pepper weevil *Anthonomus eugenii* Cano; *Anthonomus grandis* is a widespread and well-known pest of cotton. *Anthonomus eugenii* is widely distributed in the Southeastern United States, Hawaii, Mexico, Central America and the Caribbean. It feeds and develops in several species of Solanaceae but is better known as a pest of peppers, *Capsicum* spp. (Clark and Burke 1996). Other pest anthonomine include: *Anthonomus signatus* (strawberry weevil), *Anthonomus nigrinus* (potato weevil), *Anthonomus musculus* (cranberry weevil), *Anthonomus pomorum* (apple blossom weevil) *Anthonomus pyri* (pear weevil), *Anthonomus fulvipes* (cherry weevil), *Anthonomus quadrigibbus* (apple curculio), and *Pseudanthonomus validus* (currant fruit weevil), (Ahmad and Burke 1972, Burke 1976, Clark and Burke 1996, Clark 1987b and Muñiz 2001).

The objective of the key presented here is to allow identification of genera of this tribe in Mexico and Central America.

## Methods

A list of genera of Anthonomini reported in Mexico and Central America was compiled from the following works: Champion (1903, 1910), Dietz (1891), Kissinger (1964), O’Brien and Wibmer (1982, 1984a, 1984b), Alonso-Zarazaga and Lyal (1999), Anderson (2002). The original description of each genus was consulted and a data matrix constructed of morphological characters.

Specimens of 1,529 adults of the tribe Anthonomini were examined from collections of the following institutions: Instituto de Ecología, A.C, Xalapa, Veracruz, Mexico. (IEXA), Facultad de Ciencias Naturales, Universidad Autónoma de Querétaro, Mexico (UAQE), Universidad Autonoma Agraria Antonio Narro, Saltillo, Coahuila, México (UAAAN), Texas A&M University Insects Collection, College Station, Texas, U.S.A. (TAMUIC).

Images of specimens of each genus were captured with the aid of a stereoscopic microscope and digital camera and processed using COMBINEZP software (Hadley 2010). PHOTOSHOP CS3® or CORELDRAW® software programs were used to highlight or draw characters cited in the key. A synopsis of each genus is provided giving: generic name, author and recorded year, total species for New World, number of species occurring in Mexico and Central America, distributions, family placement of associated plants, and bibliographic references to identify species.

### Key to genera of Anthonomini occurring in Mexico and Central America

**Table:** 

1	Tarsal claw simple, without basal process or tooth (Fig. 1)	2
–	Tarsal claw with basal process or tooth (Figs 2 and 3)	3
2	Lateral rostral groove defined near eye (Fig. 22); elytra in lateral view rounded from middle to apex (Figs 48 and 49); metafemur lacking tooth	*Epimechus*
–	Lateral rostral groove not defined near eye (Fig. 20); elytra sloped slowly from middle to apex (Fig. 41); metafemur with a ventral tooth	*Brachyogmus*
3	Procoxae separated by process of the prosternum; mesocoxae separated by a distance nearly equal to width of one coxa (Fig. 4); antennal funiculus with 6 articles (Fig. 42)	*Huaca*
–	Procoxae contiguous (Fig. 6), if separated then profemur with a large and triangular tooth (Fig. 17); mesocoxae separated by a distance less than width of one coxa; antennal funiculus with 5,6 or 7 articles	4
4	Mesocoxae narrowly separated by distance less than 0.2× the width of one mesocoxa (Fig. 5); tarsal claw with an acute tooth arising from inner margin slightly distal of base (Figs 2 and 54)	*Madgalinops*
–	Mesocoxae widely separated by distance more than 0.2× the width of one mesocoxa; tarsal claw with a stout tooth arising from base of claw (Figs 3 and 6)	5
5	Rostrum short and moderately stout, equal to or slightly shorter than length of pronotum; elytra, base of interval 3 swollen and toothed; profemur with tooth moderately large (Figs 17, 59 and 60)	*Smicraulax*
–	Rostrum slender and longer than length of pronotum (Fig. 19); elytra, interval 3 not swollen and toothed; profemur with ventral tooth various (Figs 11–13)	6
6	Antennal funiculus with 5 articles; antennal club with basal article glossy, almost glabrous; femur lacking tooth or with a single minute ventral tooth (Fig. 57)	*Neomastix*
–	Antennal funiculus with 6 or 7 articles; antennal club various; femur with a small or large ventral tooth (Figs 13–16)	7
7	Lateral rostral groove with the dorsal margin directed toward ventral margin of eye and the ventral margin directed toward ventral margin of rostrum (Fig. 24)	8
–	Lateral rostral groove directed toward middle of eye (Fig. 19)	11
8	Antennal funiculus with 7 articles; profemur with a large and triangular ventral tooth, base of tooth equal to width of the protibia; protibia stoutly expanded on inner margin at midpoint (Figs 14, 43 and 44)	*Cionomimus*
–	Antennal funiculus with 6 articles; profemur with a small ventral tooth, base of tooth less than width of the protibia; protibia only slightly expanded or if expanded, not at midpoint (Fig. 13)	9
9	Pronotum and elytra humpbacked; profemoral tooth curved toward the tibia (Fig. 13); dense and decumbent hair-like scales throughout body and anterior 2/3 of rostrum (Fig. 40)	*Botanebius*
–	Pronotum and elytra not humpbacked; profemur with triangular tooth not curved; body and rostrum vestiture various (Figs 55 and 58)	10
10	Pronotum, elytra and legs densely clothed with scales, intermixed with distinct semierect to erect scattered scales; profemoral tooth smaller than a tarsal claw; metafemur lacking tooth (Fig. 55)	*Melexerus*
–	Pronotum, elytra and legs usually with decumbent scales; profemoral tooth nearly equal in length to a tarsal claw; metafemur with a ventral tooth (Fig. 58)	*Pseudanthonomus*
11	Profemur expanded, *ca*. 2× stouter than metafemur (Figs 10 and 15); mesocoxae narrowly separated by distance *ca*. ¼ width of one coxa (Fig. 6)	12
–	Profemur expanded by less than 2× width of metafemur (Figs 11, 12 and 16); mesocoxae separated by distance more than ¼ width of one coxa	13
12	Head strongly constricted behind eyes (Fig. 18); eyes prominent, strongly convex; body densely covered with broad to elongate hair-like scales; dark rounded or triangular patch present on disc of elytra at base (Figs 25–27)	*Achia*
–	Head subconical, slightly constricted behind eyes (Fig. 21); eyes slightly to moderately convex; vestiture of dense, elongate scales which may be intermixed with semierect, erect or recumbent scales; dark subtriangular patch of scales on each elytron past middle (Figs 45–47)	*Cionopsis*
13	Pro- and mesofemur with an anterior emargination (Figs 9 and 16); profemoral tooth slightly serrate distal to the emargination; elytral disk and declivital area covered by dense, pallid scales (Figs 52 and 53); mesotrochanters trapezoidal (Fig. 7)	*Loncophorus*
–	Pro- and mesofemur lacking emargination, if present, profemoral tooth not serrate and protibia curved (Fig. 11); elytra various (Figs 28–39); mesotrochanter triangular (Fig. 8) (*Anthonomus* subgenus *Anthonomorphus* males have a trapezoidal shaped mesotrochanter)	14
14	Rostrum densely covered with broad scales to near apex (Fig. 23); eyes small, diameter of each eye nearly equal to width of rostrum at base, slightly or strongly free behind (Fig. 56)	*Narberdia*
–	Rostrum with scales limited to basal half of length; eyes moderately large, diameter slightly or much greater than rostrum at base	15
15	Body with smooth and shining integument with scattered, narrow, white scales; posteromedian sides of pronotum straight; elytra strongly convex dorsomedially or posteromedially, sides convergent to apices (Figs 50 and 51)	*Lonchophorellus*
–	Body vestiture and shape variable; posteromedian sides of pronotum curved; elytra not strongly convex and apical sides rounded (Figs 28–39)	16
16	Elytra usually with a transverse basal patch of black scales (Figs 36, 37 and 39); subbasal, anteromedian or posteromedian elevation on even-numbered interstriae usually well-developed; profemur strongly expanded 1.5× wider than metafemur; protibia curved, apical half of inner margin expanded and carinate (Figs 12, 35–39)	*Atractomerus*
–	Elytra lacking a transverse basal patch of black scales (Figs 29 and 33); elytral elevation if present limited to odd-numbered interstriae; profemur if 1.5× wider than metafemur then, profemoral tooth usually with shallow to deep anterior emargination (Fig. 11); protibia usually straight and inner margin various (Figs 28–34)	*Anthonomus*

**Figures 4–6.**
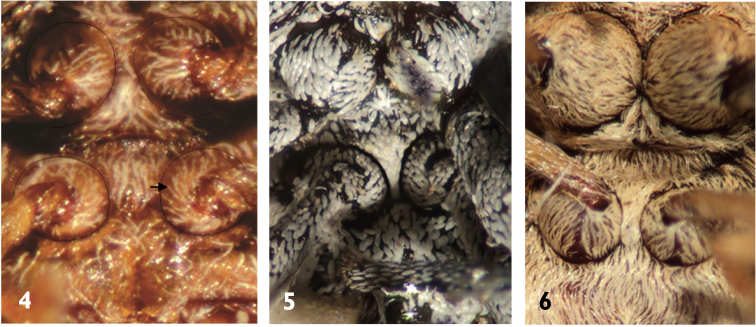
Pro- and mesocoxae (→) of Anthonomini species: **4** *Huaca mudca* **5** *Magdalinops vittipennis* **6** *Achia rhombifera*.

**Figures 7–8.**
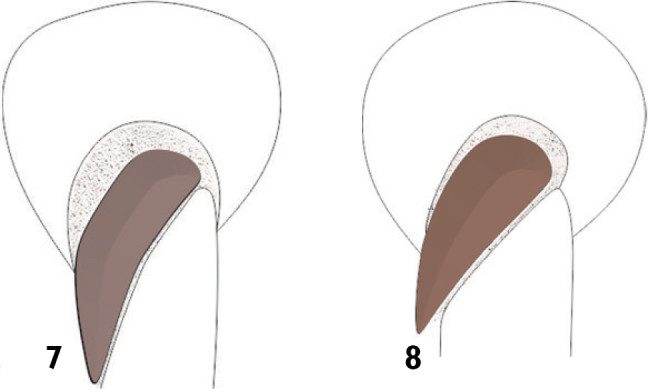
Mesotrochanter shape of two Anthonomini genera: **7** Trapezoidal, *Loncophorus* sp. **8** Triangular, *Anthonomus* sp.

**Figure 9.**
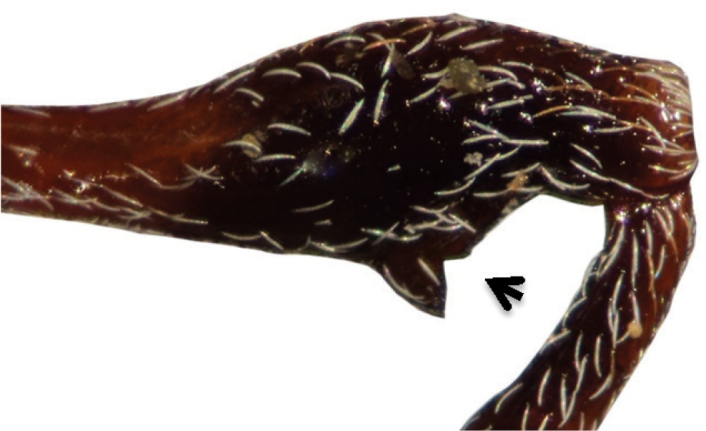
Mesofemoral tooth with an acute emargination, *Loncophorus pustulatus*.

**Figures 10–17.**
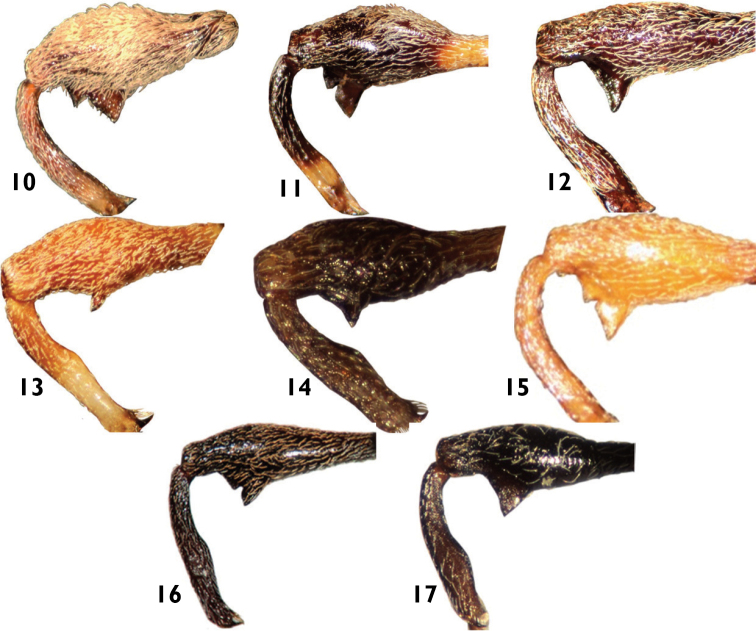
Profemora and protibiae of Anthonomini species: **10** *Achia rhombifera* **11** *Anthonomus flavirostris* **12** *Atractomerus nigrocalcaratus* **13** *Botanebius gibbosus* **14** *Cionomimus brevis* **15** *Cionopsis lineolata* **16** *Loncophorus crossi* **17** *Smicraulax nigrinus*.

**Figures 18–24.**
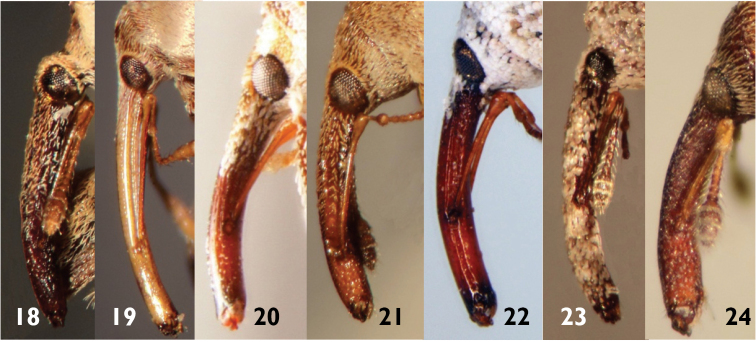
Rostra of Anthonomini species: **18** *Achia rhombifera* **19** *Anthonomus flavirostris* **20 ***Brachyogmus ornatus* **21** *Cionopsis lineolata* **22** *Epimechus flavirostris* **23** *Narberdia aridulus* **24** *Pseudanthonomus helvolus*.

**Figures 25–33.**
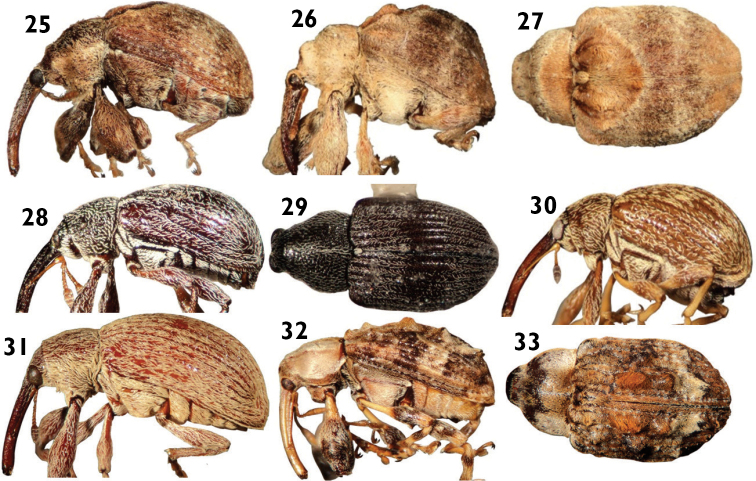
Anthonomini species: **25** *Achia serjaniae* **26** and **27** *Achia rhombifera* **28** *Anthonomus aeneolus* **29** *Anthonomus abdominalis* **30** *Anthonomus eugenii* **31** *Anthonomus grandis* **32** and **33** *Anthonomus flavirostris*.

**Figures 34–48.**
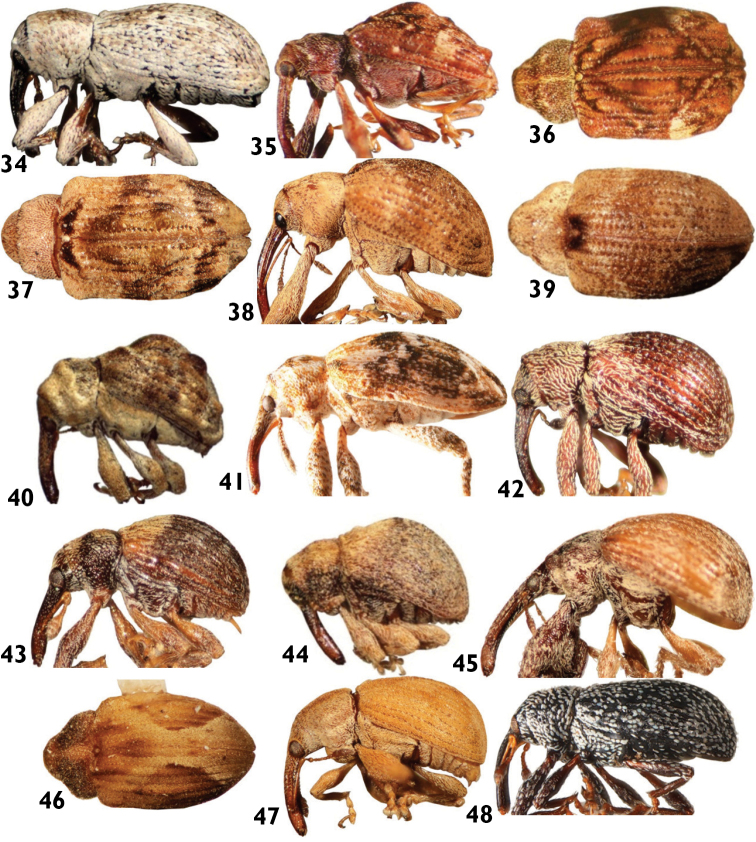
Anthonomini species: **34** *Anthonomus (Cnemocillus) tenuis* **35** and **36** *Atractomerus albolateralis* **37** *Atractomerus recessus* **38** and **39** *Atractomerus indicivus* **40** *Botanebius gibbosus* **41** *Brachyogmus ornatus* **42** *Huaca mayu* **43** *Cionomimus championi* **44** *Cionomimus insolens* **45** *Cionopsis crispula* **46 ***Cionopsis maculata* **47** *Cionopsis lineolata* **48** *Epimechus hesperius*.

**Figures 49–60.**
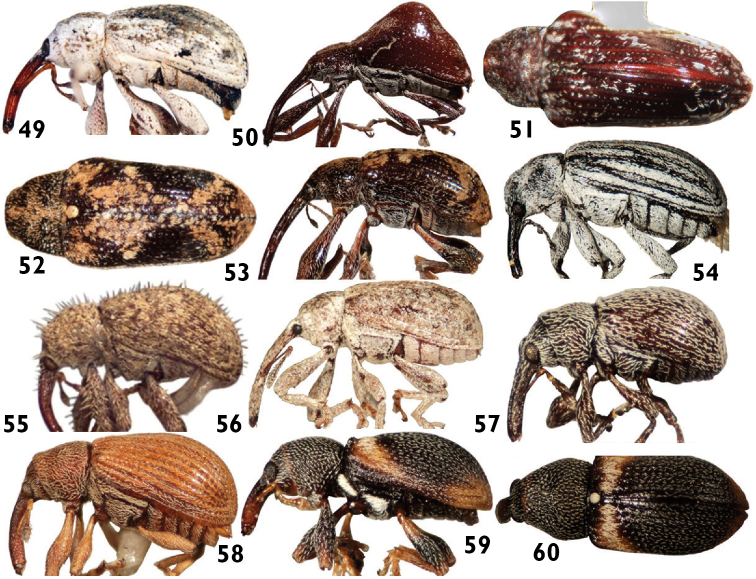
Anthonomini species: **49** *Epimechus flavirostris* **50** *Lonchophorellus scyla* **51** *Lonchophorellus callosus* **52** *Loncophorus crossi* **53** *Loncophorus pustulatus* **54** *Magdalinops vittipennis* **55** *Melexerus hispidus* **56** *Narberdia aridulus* **57** *Neomastix spatium* **58** *Pseudanthonomus helvolus* **59** and **60** *Smicraulax tuberculatus*.

## Discussion

The tribe Anthonomini (*sensu* Alonso-Zarazaga and Lyal 1999) was reviewed and delimited by Burke (1976) who considered the systematics of the taxon to be in a “chaotic” state at the tribal and lower levels. From 1976 to the present, Horace R. Burke and Wayne E. Clark have conducted revisionary taxonomic studies on many of the New World genera and species. These taxonomic publications have greatly advanced the systematics of Anthonomini for the region, although much still remains to be resolved within the tribe, especially within the genus *Anthonomus* as there are currently no keys to the various species groups recognized in the studies of Clark. The key presented here allows for the identification of the genera of the tribe reported from Mexico and Central America. Some genera can easily be either recognized by characters or combinations of characters, such as: *Brachyogmus*, *Magdalinops*, *Narberdia*, *Smicraulax* and *Huaca*; however, the intergeneric relationships of several of the species of *Anthonomus, Atractomerus, Loncophorus* and *Lonchophorellus* are not so clearly delimited and may be harder to separate.

### Synopsis of Genera list of Mexican and Central America Anthonomini

***Achia*** Champion, 1903. New World species 19, with 6 species from Mexico and Central America. Distribution: Bolivia, Brazil, Honduras, Mexico and Panama. States of Mexico: Chiapas, Mexico, Guerrero, Michoacan, Morelos, Nayarit, Oaxaca, San Luis Potosí, Tamaulipas and Veracruz. Families of associated plants include: Sapindaceae, Mimosoideae, Lauraceae, Bromeliaceae. See Burke and Kovarik (1986) and Clark et al. (2007) to separate species.

***Anthonomus*** Germar, 1817. New World species 491, with 172 species from Mexico and Central America. Distribution: worldwide in all geographical regions except Antarctica. Associated plant families include: Asteraceae, Combretaceae, Cupressaceae, Euphorbiaceae, Fabaceae, Juglandaceae, Kramericeae, Malpighiaceae, Malvaceae, Myrtaceae, Rosaceae, Rutaceae, Rhizophoraceae, Sapindaceae, Solanaceae and Vitaceae. See Burke 1962, 1964, 1979, 1984, Burke and Cate 1979, Clark 1987a, 1987c, 1987d, 1988a, 1988b, 1989b, 1990a, 1990b, 1990c, 1990d, 1991a, 1991b, 1991c, 1992a, 1992b, 1993b, 1993c, 1993d, 1994, Clark and Burke 1985, 1986a, 1986b, 1996, 2005 and Jones and Burke 1997 to separate species.

***Atractomerus*** Duponchel and Chevrolat, 1849. New World species 45, with 17 species from Mexico and Central America. Distribution: Bolivia, Brazil, French Guyana, Costa Rica, Guatemala, Mexico and Panamá; states of Mexico: Chiapas, Mexico, San Luis Potosí, Tabasco, Tamaulipas and Veracruz. Associated plant families include: Myrtaceae and Melastomataceae. See Clark (1989c) to separate species.

***Botanebius*** Schoenherr, 1836. New World species 2, with 1 species from Mexico and Central America. Distribution: Colombia, Venezuela, Cuba, Belize, Honduras, Mexico and Panama; states of Mexico: Chiapas. Associated plant is unknown.

***Brachyogmus*** Linell 1897. New World species 1, monotypic genus, *Brachyogmus ornatus* Linell 1897. Distribution: United States of America and Mexico; states of Mexico: Sonora. The species has been associated with Solanaceae Burke (1968).

***Cionomimus*** Marshall, 1939. New World species 11, with 9 species from Mexico and Central America. Distribution: Colombia, Venezuela, Guatemala, Mexico and Panama; states of Mexico: Baja California, Coahuila, Chihuahua, Chiapas, Durango, Guerrero, Hidalgo, Jalisco, Michoacán, Querétaro, Oaxaca, Nuevo León and Veracruz. Species have been associated with Santalaceae. See Burke (1981a) and Anderson (1994) to separate species.

***Cionopsis*** Champion, 1903. New World species 5, with 3 species from Mexico and Central America. Distribution: Venezuela, Guatemala, Mexico, Panama; states of Mexico: Chiapas, Guerrero, Jalisco, Morelos, Sinaloa and Veracruz. Species have been associated with Sapindaceae. See Burke (1981b) to separate species.

***Epimechus*** Dietz, 1891. New World species 11, with 7 species from Mexico. Distribution: United States of America and Mexico; States of Mexico: Baja California Coahuila, Durango, Oaxaca, Michoacan, Nayarit and Nuevo Leon. Species have been associated with Asteraceae. See Clark and Burke (2001) to separate species.

***Huaca***Clark, 1993**.** New World species 26, with 11 species from Mexico and Central America**.** Distribution: United States of America (Florida), Belize, Costa Rica, Cuba, Dominica, Honduras, Jamaica, Mexico, Panamá, Puerto Rico, Saint Christopher, Virgin Islands, Brazil, Trinidad, Uruguay, Venezuela; states of Mexico: Chiapas, Oaxaca, Quintana Roo and Tamaulipas. Families of associated plants include: Malpighiaceae, Myrtaceae, Phytolaccaceae, Rhizophoraceae, Rubiaceae and Rutaceae. See Clark (1993a) to separate species.

***Loncophorus*** Chevrolat, 1832. New World species 14, with 8 species from Mexico and Central America. Distribution: Argentina, Brazil, Colombia, Ecuador, French Guyana, Paraguay, Peru Surinam, Cuba, Costa Rica, Nicaragua, Mexico, Panama; states of Mexico: Oaxaca and Veracruz. Species have been associated with Bombacaceae. See Clark (1988c, 1995) to separate the species.

***Lonchophorellus*** Clark, 1989. New World species 4, with 2 species from Mexico and Central America. Distribution: Bolivia, Brazil, Colombia, Ecuador, Peru, Venezuela, Costa Rica, El Salvador, Guatemala, Honduras, Mexico and Panama; states of Mexico: Chiapas, Guerrero, Morelos, Puebla and Veracruz. Individuals have been associated with: Flacourtiaceae, Malpighiaceae, Myrtaceae and Sterculiaceae. See Clark (1989a) to separate species.

***Magdalinops*** Dietz, 1891. New World species 4, with 1 species from Mexico. Distribution: United States of America and Mexico; states of Mexico: Baja California. Individuals have been associated with Asteraceae. See Clark and Burke (2002) to separate species.

***Melexerus*** Burke, 1982. New World species 1, monotypic genus, *Melexerus hispidus* Burke 1982. Distribution: Colombia, El Salvador, Guatemala, Jamaica, Mexico, Venezuela; states of Mexico: Mexico, Michoacan, Morelos, Nayarit, San Luis Potosí, Sinaloa and Tamaulipas. Species has been associated with Fagaceae (Burke 1982).

***Narberdia*** Burke, 1976. New World species 1, monotypic genus, *Narberdia aridulus* Burke 1976. Distribution: United States of America and Mexico; states of Mexico: Nuevo León. Species has been associated with Euphorbiaceae (Burke and Rector 1976).

***Neomastix*** Dietz, 1891. New World species 10, with 4 species from Mexico and Central America. Distribution: Colombia, Brazil, Costa Rica, Cuba, El Salvador, United States of America, Guatemala, Honduras, Haiti, Island Virgin, México, Nicaragua, Puerto Rico; states of Mexico: Guerrero, Morelos, Oaxaca, Puebla, Quintana Roo, Sonora, Tamaulipas. Families of associated plants include: Asteraceae, Ericaceae, Fabaceae, Palmaceae, Sapindaceae and Sterculiaceae. See Clark (1993e) to separate species.

***Pseudanthonomus*** Dietz, 1891. New World species 35, with 14 species from Mexico and Central America. Distribution: Canada, Costa Rica, El Salvador, United States of America, Guadeloupe, Guatemala, Mexico, Panama and Venezuela; states of Mexico: Baja California and Baja California Sur, Chiapas, Durango Guanajuato Jalisco, Nayarit, Nuevo León, Puebla, Quintana Roo, San Luis Potosí, Tabasco Tamaulipas and Veracruz. Families of associated plants include: Betulaceae, Boraginaceae, Caprifoliaceae, Ericaceae, Hamamelidaceae, Krameriaceae, Malpighiaceae, Malvaceae, Rosaceae Saxifragaceae and Verbenaceae. See Clark (1987b, 1990e) to separate species.

***Smicraulax*** Pierce, 1908. New World species 6, 6 in Mexico and Central America. Distribution: United States of America, Guatemala, Honduras, Mexico and Panama: states of Mexico: Chiapas, Durango, Guerrero, Oaxaca and Nuevo León. Species have been associated with Santalaceae. See Burke (1975), Burke and Hafernik (1971) and Anderson (1994) to separate species.
